# Clinical Aspects of Feline Retroviruses: A Review

**DOI:** 10.3390/v4112684

**Published:** 2012-10-31

**Authors:** Katrin Hartmann

**Affiliations:** Medizinische Kleintierklinik, LMU University of Munich, Germany, Veterinaerstrasse 13, 80539 Munich, Germany; Email: hartmann@uni-muenchen.de; Tel.: +49-89-2180-2653, Fax: +49-89-2180-16501.

**Keywords:** feline leukemia virus, FeLV, feline immunodeficiency virus, FIV, clinical signs, immunosuppression, immune-mediated diseases, tumors, neurologic signs, bone marrow suppression

## Abstract

Feline leukemia virus (FeLV) and feline immunodeficiency virus (FIV) are retroviruses with global impact on the health of domestic cats. The two viruses differ in their potential to cause disease. FeLV is more pathogenic, and was long considered to be responsible for more clinical syndromes than any other agent in cats. FeLV can cause tumors (mainly lymphoma), bone marrow suppression syndromes (mainly anemia), and lead to secondary infectious diseases caused by suppressive effects of the virus on bone marrow and the immune system. Today, FeLV is less commonly diagnosed than in the previous 20 years; prevalence has been decreasing in most countries. However, FeLV importance may be underestimated as it has been shown that regressively infected cats (that are negative in routinely used FeLV tests) also can develop clinical signs. FIV can cause an acquired immunodeficiency syndrome that increases the risk of opportunistic infections, neurological diseases, and tumors. In most naturally infected cats, however, FIV itself does not cause severe clinical signs, and FIV-infected cats may live many years without any health problems. This article provides a review of clinical syndromes in progressively and regressively FeLV-infected cats as well as in FIV-infected cats.

## 1. Introduction

Feline leukemia virus (FeLV) and feline immunodeficiency virus (FIV) belong to the most common infectious diseases in cats. Both are retroviruses, but FeLV is a γ-retrovirus, while FIV is classified as a lentivirus. Although FeLV and FIV are closely related, they differ in their potential to cause disease. In the United States, prevalence of both infections is about 2% in healthy cats and up to about 30% in high-risk or sick cats [1,2]. Risk factors for infection include male gender, adulthood, and outdoor access [3,4]. Retroviral tests can diagnose only infection, not clinical disease.

FeLV is more pathogenic than FIV. For a long time, FeLV was considered to account for most disease-related deaths, and to be responsible for more clinical syndromes than any other single agent in cats. It was proposed that approximately one third of all tumor-related deaths in cats were caused by FeLV, and an even greater number of cats died of FeLV-related anemia and secondary infections caused by suppressive effects of the virus on bone marrow and the immune system. Today, these statements have to be revised, as in recent years the prevalence and consequently the importance of FeLV as a pathogen in cats have been decreasing. Still, if present in closed households with other viruses, such as feline coronavirus (FCoV), or FIV, FeLV infection has the greatest impact on survival [5]. The death rate of progressively FeLV-infected cats in multi-cat households has been estimated at approximately 50% in two years and 80% in three years [6,7], but is much lower today, at least for cats that are well taken care of and that are kept strictly indoors in single-cat households. A survey in the United States compared the survival of more than 1000 FeLV-infected cats to more than 8000 age- and sex-matched uninfected control cats and found that in FeLV-infected cats median survival was 2.4 years compared to 6.0 years for control cats [2]. Despite the fact that progressive FeLV infection is associated with a decrease in life expectancy, many owners elect to provide treatment for their cats, and with proper care, FeLV-infected cats might live for many years with good quality of life.

Although FIV can cause an acquired immunodeficiency syndrome in cats (“feline AIDS”) comparable to human immunodeficiency virus (HIV) infection in humans, with increased risk for opportunistic infections, neurologic diseases, and tumors, in most naturally infected cats, FIV does not cause a severe clinical syndrome. With proper care, FIV-infected cats can live many years and, in fact, can die at older age from causes unrelated to their FIV infection. In a follow-up study in naturally FIV-infected cats, the rate of progression was variable, with death occurring in about 18% of infected cats within the first two years of observation (about five years after the estimated time of infection). An additional 18% developed increasingly severe disease, but more than 50% remained clinically asymptomatic during the two years [8]. FIV infection has little impact on a cat population and does not reduce the number of cats in a household [2]. Thus, overall survival time is not shorter than in uninfected cats, and quality of life is usually fairly high over an extended period of time.

## 2. Stages of Infection

Both infections are chronic in nature and develop through different disease stages. Characteristically for both infections, there is a long asymptomatic phase, in which cats do not show clinical signs.

FeLV infection has different stages. Recently, novel diagnostic tools, including very sensitive PCR methods providing new data on the course of FeLV infection, have questioned the traditional understanding of FeLV pathogenesis. Cats believed to be immune to FeLV after infection were found to remain provirus-positive. Antigen-negative, provirus-positive cats are frequently detected and their clinical relevance and role in FeLV epidemiology is still not fully understood. Antigen-negative, provirus-positive cats are considered FeLV carriers. Following reactivation, they can act as an infection source. As FeLV provirus is integrated into the cat’s genome, it is unlikely to be fully cleared over time. Antigen-negative, provirus-positive cats do not shed the virus, but reactivation with reoccurring virus shedding is possible [9,10]. Based on this information, a new classification has been proposed, in which the stages of FeLV infection are defined as abortive infection (comparable to the former “regressor cats”), regressive infection (comparable to the former “transient viremia” followed by “latent infection”), progressive infection (comparable to the former “persistent viremia”), and focal or atypical infection (Table 1) [11,12,13,14].

**Abortive infection**. After infection, the virus starts initially to replicate in the local lymphoid tissue in the oropharyngeal area. In some immunocompetent cats (formerly called “regressor cats)”, viral replication may be terminated by an effective humoral and cell-mediated immune response; these cats never become viremic. They have high levels of neutralizing antibodies. Neither FeLV antigen nor viral RNA or proviral DNA can be detected in the blood at any time. Abortive infection is likely caused when a cat is exposed to low doses of FeLV [15]. It is still unknown, how often this situation really occurs in nature, because studies using very sensitive PCR methods have found that in many of the formerly considered “regressor cats”, the virus actually can still be found later in these cats when investigating tissue samples. Thus, it appears likely that none or only very few cats can completely clear FeLV infection from all cells.

**Regressive infection** develops following an effective immune response. In regressive infection, virus replication and viremia are contained prior to or shortly after bone marrow infection. After initial infection, replicating FeLV spreads systemically through infected mononuclear cells (lymphocytes and monocytes). During this stage, cats have positive results on tests that detect free antigen in plasma (e.g., ELISA). They shed virus, mainly with saliva. In cats with regressive infection, this viremia, however, is terminated within weeks or months (therefore formerly called “transient viremia”). In some cats, viremia may persist longer than three weeks. After about three weeks of viremia, bone marrow cells become infected, and infected hematopoietic precursor cells develop into infected granulocytes and platelets that circulate in the body. Even if bone marrow cells become infected, a certain percentage of cats is able to clear viremia. However, they cannot completely eliminate the virus from the body, even if they terminate viremia because the information for virus replication (proviral DNA) is present in bone marrow stem cells. This condition has been called “latent infection” (and is now a part of the regressive infection). The molecular basis of latency is the integration of a copy of the viral genome (provirus) into cellular chromosomal DNA. Although proviral DNA remains present within the cellular genome, no virus is actively produced. Thus, cats with regressive infection have negative results in all tests that detect FeLV antigen. During cell division, proviral DNA is replicated and the information given to the daughter cells. Thus, complete cell lineages may contain FeLV proviral DNA. However, proviral DNA is not translated into proteins, and no infectious virus particles are produced. Therefore, regressively infected cats do not shed FeLV and are not infectious to others. Sensitive PCR methods can detect provirus in the blood of cats with regressive infection that are antigen-negative. In a study in Switzerland it was shown that in addition to the antigen-positive, provirus-positive cats, about 10% of the cat population that were negative for antigen were positive for proviral DNA in the blood [16]. Regressive infection can be reactivated because the information for producing complete viral particles is present and can potentially be reused when antibody production decreases (e.g., after immunosuppression).

In cats with **progressive infection**, FeLV infection is not contained early in the infection. Thus, extensive virus replication occurs, first in the lymphoid tissues, followed by the bone marrow and mucosal and glandular epithelial tissues. Progressively infected cats remain persistently viremic. They are infectious to other cats for the remainder of their life. This condition has been called “persistent viremia” and is now classified as progressive infection. Cats with progressive infection develop FeLV-associated diseases, and most of them will die within a few years. Regressive and progressive infections can be distinguished by repeated testing for viral antigen in peripheral blood; regressively infected cats will turn negative at latest 16 weeks after infection, while progressively infected cats will remain positive. Initially both, regressive and progressive infections are accompanied by persistence of FeLV proviral DNA in the blood detected by PCR but later are associated with different FeLV loads when measured by quantitative PCR; regressive infection is associated with low, progressive infection with high virus load [11,17].

**Focal infections** or atypical infections have been reported in up to 10% of experimentally infected cats. Focal infections or atypical infections may also be observed in natural infections, but are probably rare in the field. Focal infections are characterized by a persistent atypical local viral replication (e.g., in mammary glands, bladder, eyes). This replication can lead to intermittent or low-grade production of antigen, and therefore, these cats can have weakly positive or discordant results in antigen tests, or positive and negative results may alternate [14].

**Table 1:** Stages of feline leukemia virus (FeLV)infection

Stages of FeLV infection	FeLV p27 antigen in blood	Virus blood culture	Viral RNA in blood	Viral DNA in blood	Viral tissue culture	Viral shedding	FeLV-associated disease
**Progressive**	Positive	Positive	Positive	Positive	Positive	Positive	Likely
**Regressive**	Negative	Negative	Negative	Positive	Negative	Negative	Unlikely
**Abortive**	Negative	Negative	Negative	Negative	Negative	Negative	Unlikely
**Focal**	Variable	Variable	Variable	Variable	Positive	Variable	Unlikely

Experimental FIV infection also progresses through several stages, similar to HIV infection in people, including an acute phase, a clinically asymptomatic phase of variable duration, and a terminal phase sometimes called “feline acquired immunodeficiency syndrome” (“AIDS”) [18,19]. However, there is no clear distinction between these stages in naturally FIV-infected cats, and not all stages are apparent; therefore, the usefulness of this staging in natural FIV infection has been questioned. Moreover, even cats in moribund condition with severe immunosuppression and secondary infections may fully recover with appropriate care and return to an asymptomatic stage. Thus, different from HIV-infected people, cats classified as being in the “AIDS phase” (high virus load, severe clinical signs due to secondary infection) can recover and be asymptomatic again, and their virus loads can even decrease dramatically.

## 3. Clinical Signs

Clinical signs in both retrovirus infections are variable. After a long asymptomatic phase, cats can develop tumors, hematopoietic disorders, neurologic disorders, immunodeficiency, immune-mediated diseases, and stomatitis. The pathomechanism of these disorders is different in both retrovirus infections (Table 2).

Although FeLV was named after a tumor that first garnered its attention, most infected cats are presented to the veterinarian not for tumors but for anemia or immunosuppression. Clinical signs associated with FeLV infection can be classified as tumors, immunosuppression, hematologic disorders, immune-mediated diseases, and other syndromes (including neuropathy, reproductive disorders, fading kitten syndrome). Of 8642 FeLV-infected cats presented to North American Veterinary Teaching Hospitals, various co-infections (including FIV infection, feline infectious peritonitis (FIP), upper respiratory infection, hemotropic mycoplasmosis, and stomatitis) were the most frequent findings (15%), followed by anemia (11%), lymphoma (6%), leukopenia or thrombocytopenia (5%), and leukemia or myeloproliferative diseases (4%) [20]. The outcome of FeLV infection and the clinical course are determined by a combination of viral and host factors. Some of the differences in outcome can be traced to properties of the virus itself, such as the subgroup that determines differences in the clinical picture (e.g., FeLV-B is primarily associated with tumors, FeLV-C is primarily associated with non-regenerative anemia). A study aiming to define dominant host immune effects or mechanisms responsible for the outcome of infection by using longitudinal changes in FeLV-specific cytotoxic T-lymphocytes (CTL) found that high levels of circulating FeLV-specific effector CTLs appear before virus-neutralizing antibodies in cats that have recovered from exposure to FeLV. In contrast, progressive infection with persistent viremia has been associated with a silencing of virus-specific humoral and cell-mediated immunity host effector mechanisms [21]. Probably the most important host factor that determines the clinical outcome of cats infected with FeLV is the age of the cat at the time of infection [22]. Neonatal kittens develop marked thymic atrophy after infection (“fading kitten syndrome”), resulting in severe immunosuppression, wasting, and early death. As cats mature, they acquire a progressive resistance. When older cats become infected, they tend to have abortive or regressive infections or, if developing progressive infection, have at least milder signs and a more protracted period of apparent good health [7].

Clinical signs in naturally FIV-infected cats usually reflect secondary diseases, such as infections and neoplasia, to which FIV-infected cats are considered more susceptible. FIV itself may cause some clinical features (e.g., neurologic signs) resulting from abnormal function or inflammation of affected organs. In experimental infection, an initial stage is sometimes noticed usually with transient and mild clinical signs, including fever, lethargy, signs of enteritis, stomatitis, dermatitis, conjunctivitis, respiratory tract disease, and generalized lymph node enlargement [23]. The acute phase may last several days to a few weeks, after which cats will enter a period in which they appear clinically healthy. This phase is usually not noticed by the owners in naturally infected cats. The duration of the following asymptomatic phase varies, but usually lasts many years. Factors that influence the duration of the asymptomatic phase include the pathogenicity of the infecting isolate (also depending on the FIV subtype), exposure to secondary pathogens, and the age of the cat at the time of infection [24,25]. In the last, symptomatic phase (“AIDS phase”) of infection, the clinical signs are a reflection of opportunistic infections, neoplasia, myelosuppression, and neurologic disease.

### 3.1. Tumors

While FeLV-infected cats are 62-times more likely to develop lymphoma or leukemia than non-infected cats and FeLV plays a direct role in tumorgenesis, FIV-infected cats have about a five-fold increased risk of tumor development, and the role of FIV is usually indirect. Lymphomas are the most common tumors in FeLV- and FIV-infected cats. While FeLV-infected cats have most commonly T-cell lymphomas, lymphomas in FIV are mostly of B-cell origin [26,27].

FeLV is a major oncogene that causes different tumors in cats, most commonly lymphoma and leukemia, less often other hematopoietic tumors and rarely other malignancies (including neuroblastoma, osteochondroma, and others). The association between FeLV and lymphomas has been clearly established in several ways. First, these malignancies can be induced in kittens by experimental FeLV infection [28,29,30]. Second, cats naturally infected with FeLV have a higher risk of developing lymphoma than uninfected cats [29,31]. Third, most cats with lymphoma were—at least in earlier times when prevalence of FeLV was still higher—FeLV-positive in tests that detected infectious virus or FeLV antigens. Previously, up to 80% of feline lymphomas and leukemias were reported to be FeLV-related [32,33,34,35,36,37,38]. However, since the 1980s a reduction in the prevalence of viremia has been noted in cats with lymphoma [39,40,41]. The decrease in prevalence of FeLV infection in cats with lymphoma or leukemia also indicates a shift in tumor causation in recent years. Whereas 59% of all cats with lymphoma or leukemia were FeLV antigen-positive in one German study from 1980 to 1995, only 20% of the cats were FeLV antigen-positive in the years 1996 to 1999 in the same University Teaching Hospital [41]. In a recent study in the Netherlands, only four of 71 cats with lymphoma were FeLV-positive, although 22 of these cats had mediastinal lymphoma, which previously was strongly associated with FeLV infection [42]. A greater prevalence of lymphoma in older-age cats in now observed. The major reason for the decreasing association of FeLV with lymphoma is the decreasing prevalence of FeLV infection in the overall cat population as a result of FeLV vaccination as well as testing and elimination programs. However, prevalence of lymphomas caused by FeLV may be higher than indicated by conventional antigen testing of blood [43]. Cats from FeLV cluster households had a 40-fold higher rate of development of FeLV-negative lymphoma than did those from the general population. FeLV-negative lymphomas have also occurred in laboratory cats known to have been infected previously with FeLV [44]. FeLV proviral DNA was detected in lymphomas of cats that tested negative for FeLV antigen [43], also suggesting that the virus may be associated with a larger proportion of lymphomas than previously thought. FeLV has been shown to incorporate cellular genes; several such transducted genes also present in regressively infected cells have been implicated in viral oncogenesis [44,45,46]. It is still unclear, how common regressive FeLV infection is responsible for FeLV-associated tumors in the field as study results have been controversial. Proviral DNA was detected in formalin-fixed, paraffin-embedded tumor tissue in 7/11 FeLV-negative cats with lymphoma [43]. However, other groups found evidence of provirus in only 1/22 [45] and in 0/50 FeLV antigen-negative lymphomas [47].

The most important mechanism by which FeLV causes malignancy is by insertion of the FeLV genome into the cellular genome near a cellular oncogene (most commonly *myc*), resulting in activation and over-expression of that gene. These effects lead to uncontrolled proliferation of these cells (clone). A malignancy results in the absence of an appropriate immune response. FeLV may also incorporate the oncogene to form a recombinant virus (e.g., FeLV-B, FeSV) containing cellular oncogene sequences that are then rearranged and activated. When they enter a new cell, these recombinant viruses are oncogenic. In a study of 119 cats with lymphomas, transduction or insertion of the *myc* locus had occurred in 38 cats (32%) [48]. Thus, FeLV-induced neoplasms are caused, at least in part, by somatically acquired insertional mutagenesis in which the integrated provirus may activate a proto-oncogene or disrupt a tumor suppressor gene. A recent study suggested that the *U3*-*LTR* region of FeLV transactivates cancer-related signaling pathways through production of a non-coding 104 base RNA transcript that activates NF kappaB [49]. Twelve common integration sites for FeLV associated with lymphoma development have been identified in six loci: *c-myc*, *flvi-1*, *flvi-2* (contains *bmi-1*), *fit-1*, *pim-1*, and *flit-1*. Oncogenic association of the loci is based on the fact that *c-myc* is known as a proto-oncogene, *bmi-1* and *pim-1* have been recognized as *myc*-collaborators, *fit-1* appears to be closely linked to *myb*, and *flit-1* insertion was shown to be associated with over-expression of cellular genes, e.g., *activin-A receptor type II-like 1* (*ACVRL1*). [50]. *Flit-1* seems to have an important role in the development of lymphomas and appears to represent a common novel FeLV proviral integration domain that may influence lymphomagenesis by insertional mutagenesis. Among 35 FeLV-related tumors, 5/25 thymic lymphomas demonstrated proviral insertion within *flit-1 *locus, whereas 0/4 alimentary lymphomas, 5/5 multicentric lymphomas, and 1/1 T-lymphoid leukemia examined had rearrangements in this region. Expression of *ACVRL1* mRNA was detected in the two thymic lymphomas with *flit-1* rearrangement, whereas normal thymuses and seven lymphoid tumors without *flit-1* rearrangement had no detectable *ACVRL1* mRNA expression [51].

Fibrosarcomas that are associated with FeLV are caused by FeSV, a recombinant virus that develops *de novo* in FeLV-A-infected cats by recombination of the FeLV-A genome with cellular oncogenes. Through a process of genetic recombination, FeSV acquires one of several oncogenes, such as *fes*, *fms*, or *fgr*. As a result, FeSV is an acutely transforming (tumor-causing) virus, leading to a polyclonal malignancy with multifocal tumors arising simultaneously after a short incubation period. With the decrease in FeLV prevalence, FeSV also has become less common. FeSV-induced fibrosarcomas are multicentric and usually occur in young cats. Strains of FeSV identified from naturally occurring tumors are defective and unable to replicate without the presence of FeLV-A as a helper virus that supplies proteins (such as those coded by the *env* gene) to FeSV. Fibrosarcomas caused by FeSV tend to grow rapidly, often with multiple cutaneous or subcutaneous nodules that are locally invasive and metastasize to the lung and other sites. Solitary fibrosarcomas in older cats are not caused by FeSV. These tumors are slower growing, locally invasive, slower metastasizing, and only occasionally curable by excision combined with radiation and/or gene therapy. They usually are classified as feline injection site sarcomas (FISS) caused by the granulomatous inflammatory reaction at the injection site, commonly occurring after inoculation of adjuvant-containing vaccines. It has been demonstrated that neither FeSV nor FeLV play any role in the development of FISS [52].

A few other tumors have been found in FeLV-infected cats; some of them might have an association with FeLV, others likely have just been observed by chance simultaneously in an infected cat. Iris melanomas, for example, are not associated with FeLV infections, although in one study three of 18 eyes tested positive for FeLV/FeSV proviral DNA [53]. In a more recent study, however, immunohistochemical staining and PCR did not find FeLV or FeSV in the ocular tissues of any cat with this disorder [54]. Multiple osteochondromas (cartilaginous exostoses on flat bones of unknown pathogenesis) have been described in FeLV-infected cats. Although histologically benign, they may cause significant morbidity if they occur in an area such as a vertebra and put pressure on the spinal cord or nerve roots [55,56]. In spontaneous feline olfactory neuroblastomas (aggressive, histologically inhomogenous tumors of the tasting and smelling epithelium of nose and pharynx with high metastasis rates), budding FeLV particles were found in the tumors and lymph node metastases, and FeLV DNA was detected in tumor tissue [57]. The exact role of FeLV in the genesis of these tumors is uncertain. Cutaneous horns are a benign hyperplasia of keratinocytes that have been described in FeLV-infected cats [58], but the role of FeLV is also unclear.

FIV-infected cats are about five times more likely to develop lymphoma or leukemia than non-infected cats [26,27]. Lymphomas (mostly B-cell lymphomas) [26,27,59,60], leukemias, but also several other tumors have been described in association with FIV infection [26,61,62,63,64,65,66], including squamous cell carcinoma, fibrosarcoma, and mast cell tumor. FIV provirus, however, is only occasionally detected in tumor cells [67,68,69,70], suggesting a more indirect role in lymphoma formation, such as decreased cell-mediated immune surveillance or chronic B-cell hyperplasia [68,71]. However, clonally integrated FIV DNA was found in lymphoma cells from one cat that had been experimentally infected six years earlier, indicating the possibility of an occasional direct oncogenic role of FIV [67,70,72]. The prevalence of FIV infection in one cohort of cats with lymphoma was 50% [60], much higher than the FIV prevalence in the population of cats without lymphomas, which is also supportive of a cause and effect relationship. FIV could alternatively increase tumor incidence by decreasing tumor immunosurveillance mechanisms. It also could promote tumor development through the immunostimulatory effects of replicating in lymphocytes.

### 3.2. Myelosuppression

Myelosupression and other hematopoietic disorders can occur in both, FeLV and FIV infection. It is, however, much more common and more severe in FeLV-infected cats.

Hematologic changes described in association with FeLV include anemia (non-regenerative or regenerative), persistent, transient, or cyclic neutropenia, platelet abnormalities (thrombocytopenia and platelet function abnormalities), aplastic anemia (pancytopenia), and panleukopenia-like syndrome. For the majority of pathogenic mechanisms in which FeLV causes bone marrow suppression, active virus replication is required. However, it has been demonstrated that in some FeLV antigen-negative cats, regressive FeLV infection without viremia may be responsible for bone marrow suppression. In a recent study including 37 cats with myelosuppression that tested FeLV antigen-negative in peripheral blood, 2/37 cats (5%) were found regressively infected with FeLV by bone marrow PCR (both had non-regenerative anemia) [73]. In these regressively infected cats, FeLV provirus may interrupt or inactivate cellular genes in the infected cells, or regulatory features of viral DNA may alter expression of neighboring genes. Additionally, cell function of provirus-containing myelomonocytic progenitor and stromal fibroblasts that provide bone marrow microenvironment may be altered. Alternatively, FeLV provirus may cause bone marrow disorders by inducing the expression of antigens on the cell surface, resulting in an immune-mediated destruction of the cell. Anemia is a major non-neoplastic complication that occurs in a majority of FeLV-infected cats [4]. Anemia in FeLV-infected cats may have various causes. Approximately 10% of FeLV-associated anemias are regenerative [74], most FeLV-associated anemias, however, are non-regenerative and are caused by the bone marrow suppressive effect of the virus resulting from primary infection of hematopoietic stem cells and infection of stroma cells that constitute the supporting environment for hematopoietic cells. *In vitro* exposure of normal feline bone marrow to some strains of FeLV caused suppression of erythrogenesis [6]. In addition to the direct effect of the virus on erythropoiesis, other factors can cause non-regenerative anemia in FeLV-infected cats (e.g., anemia of chronic inflammation promoted by high concentration of cytokines). FeLV infection can cause decreased platelet counts. It also can be responsible for platelet function deficits, and the lifespan of platelets is shortened in some FeLV-infected cats. Thrombocytopenia (resulting in bleeding disorders) can occur secondary to decreased platelet production from FeLV-induced bone marrow suppression or leukemic infiltration. Platelets harbor FeLV, and megakaryocytes are frequent targets of progressive FeLV infection. Immune-mediated thrombocytopenia, which rarely occurs as a single disease entity in cats, often accompanies immune-mediated hemolytic anemia (IMHA) in cats with underlying FeLV infection. FeLV infection also can cause decreased neutrophil or lymphocyte counts. Neutropenia is common in FeLV-infected cats [75] and generally occurs alone or in conjunction with other cytopenias. In some cases, myeloid hypoplasia of all granulocytic stages is observed, suggesting infection on neutrophil precursors. In some neutropenic FeLV-infected cats, an arrest in bone marrow maturation can occur at the myelocyte and metamyelocyte stages. It has been hypothesized that an immune-mediated mechanism is responsible in cases in which neutrophil counts recover with glucocorticoid treatment (“glucocorticoid-responsive neutropenia”).

Hematopoietic neoplasia (“myeloprolifertaive disorders”), including leukemia, can also cause bone marrow suppression syndromes by crowding out. Myelodysplastic syndrome (MDS), characterized by peripheral blood cytopenias and dysplastic changes in the bone marrow, is a pre-stage of acute myeloic leukemia. It was found that changes of the *LTR *region of the FeLV genome (presence of three tandem direct 47-bp repeats in the upstream region of the enhancer (*URE*)) are strongly associated with the induction of MDS [76]. Myelofibrosis, another cause of bone marrow suppression, is a condition characterized by abnormal proliferation of fibroblasts resulting from chronic stimulation of the bone marrow, such as chronic bone marrow activity from hyperplastic or neoplastic regeneration caused by FeLV. In severe cases, the entire endosteum within the medullary cavity can be obliterated.

Feline panleukopenia-like syndrome (FPLS), also known as FeLV-associated enteritis (FAE) or myeloblastopenia, consists of severe leukopenia (< 3000 cells/μl) with enteritis and destruction of intestinal crypt epithelium that mimics feline panleukopenia caused by feline panleukopenia virus (FPV) infection. However, FPV antigen has been demonstrated by IFA in intestinal sections of cats that died from this syndrome after being experimentally infected with FeLV [77]. FPV was also demonstrated by electron microscopy despite negative FPV antigen tests. It appears that this syndrome might actually not be caused by FeLV itself, as previously thought, but by co-infection with FPV. The syndrome also has been referred to as FAE in cats with progressive FeLV infection because the clinical signs observed are usually gastrointestinal, including hemorrhagic diarrhea, vomiting, oral ulceration or gingivitis, anorexia, and weight loss [78,79]. It is still unclear whether all theses syndromes have the same origin and are simply caused by co-infection with FPV (and even modified life FPV vaccines have been discussed) or if they are caused by FeLV itself [77].

Although cytopenias caused by bone marrow suppression are a common finding in FeLV infection, these are rather uncommon in FIV-infected cats. During the acute phase of infection, FIV-infected cats can exhibit mild neutropenia, which resolves as the cat progresses to the asymptomatic phase of infection. Clinically ill FIV-infected cats in a later phase of infection may have a variety of cytopenias, with lymphopenia being most common. Lymphopenia is caused by direct replication of the virus in CD4^+^ lymphocytes. Anemia and neutropenia (usually mild) may also be seen [4,51], although these abnormalities may be as much a reflection of concurrent disease as direct effects of FIV itself. A recent study in a high number (3784) of client-owned field cats compared hematologic parameters in FIV-infected, FeLV-infected and uninfected cats [4]. Anemia and thrombocytopenia were not significantly more common in FIV-infected *versus* uninfected cats. Only neutropenia was significantly more often present, in about 25% of FIV-infected cats. Soluble factors have been shown to inhibit bone marrow function in FIV-infected cats, and bone marrow infection has been associated with decreased ability to support hematopoietic potential *in vitro* or has been proposed as a mechanism underlying the development of cytopenias [51].

### 3.3. Neurologic Dysfunction

Neurologic dysfunction may be present in FeLV- and in FIV-infected cats and is one of the few syndromes directly caused by the retrovirus. However, mechanisms of neurologic dysfunction are different with both viruses.

In FeLV-infected cats, most neurologic signs are caused by lymphoma and lymphocytic infiltrations in brain or spinal cord leading to compression, but in some cases, no tumor is detectable with diagnostic imaging methods or at necropsy. In these cats, FeLV-induced neurotoxicity is suspected. Anisocoria, mydriasis, central blindness, or Horner’s syndrome have been described in FeLV-infected cats without morphologic changes. In some regions (such as the southeastern United States), urinary incontinence caused by neuropathies in FeLV-infected cats has been described [80]. Direct neurotoxic effects of FeLV have been discussed as pathogenetic mechanisms. FeLV envelope glycoproteins may be able to produce increased free intracellular calcium leading to neuronal death (this has also been described in HIV-infected humans). A polypeptide of the FeLV envelope was found to cause dose-dependent neurotoxicity associated with alterations in intracellular calcium ion concentration, neuronal survival, and neurite outgrowth. The polypeptide from a FeLV-C strain was significantly more neurotoxic than the same peptide derived from a FeLV-A strain [81,82]. Neurologic signs in 16 cats with progressive FeLV infection consisted of abnormal vocalization, hyperesthesia, and paresis progressing to paralysis. Some cats developed anisocoria or urinary incontinence during the course of their illness. Others had concurrent FeLV-related problems such as myelodysplastic disease. The clinical course of affected cats involved gradually progressive neurologic dysfunction. Microscopically, white-matter degeneration with dilation of myelin sheaths and swollen axons was identified in the spinal cord and brain stem of affected animals [80]. Immunohistochemical staining of affected tissues revealed consistent expression of FeLV p27 antigens in neurons, endothelial cells, and glial cells, and proviral DNA was amplified from multiple sections of the spinal cord [80]. These findings suggest that in some FeLV-infected cats, the virus may directly affect CNS cells cytopathically.

Neurologic signs also have been described in both natural and experimental FIV infections [83,84,85,86,87,88]. About 5% of symptomatic FIV-infected cats have a neurological disease as a predominant clinical feature. Neurologic disorders in FIV infection seem to be strain-dependent [89]. Both central and peripheral neurologic manifestations have been described, comparable to the changes in HIV-infected human beings. Dementia in human patients with AIDS is often characterized by a slight decline in cognitive ability or behavior, changes that may be too subtle to be recognized in cats. Neurological abnormalities seen in naturally infected cats tend to be more behavioral than motor. Psychotic behavior, twitching movements of the face and tongue, compulsive roaming, dementia, loss of bladder and rectal control, and disturbed sleep patterns have been observed. Other signs described include nystagmus, ataxia, seizures, and intention tremors [90,91,92]. Abnormal forebrain electrical activity and abnormal visual and auditory-evoked potentials have also been documented in cats that appeared otherwise normal [24,66,93,94]. Although the majority of FIV-infected cats do not show clinically overt neurologic signs, a much higher proportion of infected cats have microscopic CNS lesions. Brain lesions may occur in the absence of massive infection, and abnormal neurologic function has been documented in FIV-infected cats with only mild to moderate histologic evidence of inflammation [8]. Pathologic findings include the presence of perivascular infiltrates of mononuclear cells, diffuse gliosis, glial nodules, and white matter pallor. These lesions are usually located in the caudate nucleus, midbrain, and rostral brain stem [8]. Mostly, abnormal neurologic function is the result of a direct effect of the virus on CNS cells. Neurologic signs upon FIV infection are highly strain-dependent. The virus infects the brain early, with virus-induced CNS lesions sometimes developing within two months of experimental infection [8]. Microglia and astrocytes are infected by FIV, but the virus does not infect neurons. However, neuronal death has been associated with FIV infection; in particular, forebrain signs are often a result of direct neuronal injury from the virus. The exact mechanism of neuronal damage by FIV is unclear but may include neuronal apoptosis, effects on the neuron supportive functions of astrocytes, toxic products released from infected microglia, or cytokines produced in response to viral infection. *In vitro* studies support the hypothesis that FIV infection may impair normal metabolism in CNS cells, particularly astrocytes [8]. Documented abnormalities of astrocyte function include altered intercellular communication, abnormal glutathione reductase activity that could render cells more susceptible to oxidative injury, and alterations in mitochondrial membrane potential that disrupt the energy-producing capacities of the cell [95]. Astrocytes are by far the most common cell type of the brain and are important in maintaining CNS neuronal vascular microenvironment. One of the most important functions of astrocytes is to regulate the level of extracellular glutamate, a major excitatory neurotransmitter that accumulates as a consequence of neuronal activity. Excessive extracellular glutamate often results in neuronal toxicity and death. FIV infection of feline astrocytes can significantly inhibit their glutamate-scavenging ability, potentially resulting in neuronal damage [95,96]. Sometimes, neurologic signs may also be caused by opportunistic infections such as toxoplasmosis, cryptococcosis, or FIP.

### 3.4. Immunodeficiency and Secondary Infections

The most clinically important consequence of both retrovirus infections is immunosuppression. Immunosuppression can lead to secondary infectious diseases accounting for most clinical signs, but also can lead to decreased tumor surveillance mechanisms causing an increased risk of tumor development. It is important to realize that many of these secondary diseases in FeLV- and FIV-infected cats are treatable. The mechanisms that cause the immunosuppression are different for the two infections.

Many FeLV-infected cats have concurrent bacterial, viral, protozoal, and fungal infections, but few controlled studies exist proving that these cats have a higher rate of infection than FeLV-negative cats. Thus, although FeLV certainly can suppress immune function, it should not be assumed that all concurrent infections are a direct consequence of FeLV infection. Progressively FeLV-infected cats develop immunosuppression similar to that in HIV-infected people. The exact mechanisms of how the virus destroys the immune system are poorly understood, as is why different animals have such varying degrees of immunosuppression. Immunosuppression has been associated with non-integrated viral DNA from replication-defective viral variants [97]. These pathogenic immunosuppressive variants, such as FeLV-T, require a membrane-spanning receptor molecule (*Pit1*) and a second co-receptor protein (*FeLIX*) to infect T lymphocytes [98]. The latter protein is an endogenously expressed protein encoded by an endogenous provirus arising from FeLV-A, which is similar to the FeLV receptor-binding protein of FeLV-B [99].

FeLV-infected cats may develop thymic atrophy and depletion of lymph node paracortical zones following infection. Lymphopenia and neutropenia are common. In addition, neutrophils of viremic cats have decreased chemotactic and phagocytic function compared with those of normal cats. In some cats, lymphopenia may be characterized by preferential loss of CD4^+^ helper T cells, resulting in an inverted CD4/CD8 ratio (as typically seen in FIV infection) [100,101], but more commonly, substantial losses of helper cells and cytotoxic suppressor cells (CD8^+^ cells) occur [101]. Many immune function tests of naturally FeLV-infected cats are abnormal, including decreased response to T-cell mitogens, prolonged allograft reaction, reduced immunoglobulin production, depressed neutrophil function, and complement depletion. IL-2 and IL-4 are decreased in some cats [7,102], but FeLV does not appear to suppress IL-1 production from infected macrophages. IFN-γ may be deficient or increased. Increased TNF-α has been observed in serum of infected cats and in infected cells in culture. Each cytokine plays a vital role in the generation of a normal immune response, and the excess production of certain cytokines, such as TNF-α, can also cause illness. T-cells of FeLV-infected produce significantly lower levels of B-cell stimulatory factors than do those of normal cats (this defect becomes progressively more severe over time) [72], but when B-cells of FeLV-infected cats are stimulated *in vitro* by uninfected T-cells, their function remains normal. Primary and secondary humoral antibody responses to specific antigens are decreased and may occur delayed in FeLV-infected cats. In vaccination studies, FeLV-infected cats were not able to mount an adequate immune response to vaccines, such as rabies. Therefore, protection in a FeLV-infected cat after vaccination is not complete and not comparable to that in a healthy cat;thus, more frequent vaccinations (*e.g.*, every six months) have to be considered.

In FIV-infected cats, immunosuppression usually occurs in later stages of the infection, and leads to predisposition for secondary infections. In a survey study of 826 naturally FIV-infected cats examined at North American Veterinary Teaching Hospitals, the most common disease syndromes were stomatitis, neoplasia (especially lymphoma and cutaneous squamous cell carcinoma), ocular disease (uveitis and chorioretinitis), anemia and leukopenia, opportunistic infections, renal insufficiency, lower urinary tract disease, and endocrinopathies, such as hyperthyroidism and diabetes mellitus [78]. Some of these problems, however, are most likely associated rather with the older age at which these cats presented (e.g., endocrinopathies, renal insufficiency) than with their FIV infection. Infections with many different “opportunistic” pathogens of viral, bacterial, protozoal, and fungal origin have been reported in FIV-infected cats. Few studies, however, have compared the prevalence of most of these infections in FIV-infected and non-infected cats, and thus, their relevancy as true secondary invaders is unclear.

The most important immunologic abnormality shown in experimental [104,105,106] as well as in natural [107,108] infection is a decrease in the number and relative proportion of CD4^+^ cells in the peripheral blood as well as in most primary lymphoid tissues [109]. Loss of CD4^+^ cells leads to inversion of the CD4/CD8 ratio. In addition, an increase in the proportion of CD8^+^ cells also contributes to the inversion [104,108,110], in particular a population referred to as “CD8^+^ alpha-hi, beta-low cells” [111,112,113], a subset of CD8^+ ^cells that may contribute to suppression of viremia in FIV-infected cats. Causes of CD4^+^ cell loss include decreased production secondary to bone marrow or thymic infection, lysis of infected cells induced by FIV itself (cytopathic effects), destruction of virus-infected cells by the immune system, or death by apoptosis (cell death that follows receipt of a membrane signal initiating a series of programmed intracellular events) [114,115,116,117,118,119,120,121,122,123,124,125,126]. The degree of apoptosis correlates inversely with the CD4^+^ numbers and the CD4/CD8 ratio [127]. FIV *env* proteins are capable of inducing apoptosis in mononuclear cells by a mechanism that requires CXCR4 binding [128]. Ultimately, loss of CD4+ cells impairs immune responses, because CD4^+^ cells have critical roles in promoting and maintaining both humoral and cell-mediated immunity. A certain subset of CD4^+^ cells, the “Treg” (for T-regulatory cells), also seems to play an important role, and Treg cells with suppressive activity have been documented during early [129] and chronic FIV infection [130]. In FIV-infected cats, increased activity of Treg cells could thus play a role in suppressing immune responses to foreign antigens or pathogens. In addition, Treg cells are themselves targets for FIV infection [129,131], and may serve as a FIV reservoir during the latent stage of infection and be capable of stimulating virus production [132]. In addition, other immunologic abnormalities can be found. Lymphocytes may lose the ability to proliferate in response to stimulation with mitogens or antigens, and priming of lymphocytes by immunogens may be impaired [105,133,134,135,136,137,138,139]. Lymphocyte function may be reduced by altered expression of cell surface molecules, such as CD4, major histocompatibility complex II antigens, or cytokines and cytokine receptors [140,141,142,143,144], or through over-expression of abnormal molecules, such as receptors [145], leading to disrupted production of cytokines or receptor function. Impaired neutrophil adhesion and emigration in response to bacterial products have been described in FIV-infected cats [146,147,148]. Natural killer cell activity may be diminished [149] or increased [150], in acutely or asymptomatically infected cats, respectively. Changes in cytokine pattern include increased production of IFN-γ, TNF-α, IL-4, IL-6, IL-10, and IL-12 [151,152,153,154], but also differences in cytokine ratios (*e.g.*, IL-10/IL-12 ratio) [155,156].

### 3.5. Immune-mediated Diseases

In addition to a dysregulation of the immune system leading to immunosuppression, retrovirus-infected cats can also develop immune-mediated diseases caused by an overactive immune response. The most commonly seen immune-mediated response is a hypergammaglobulinemia which is caused by an excessive antibody response against the chronic persistent infection. The produced antibodies are not neutralizing and thus, may lead to antigen antibody complex formation. These immune complexes can deposit, usually in narrow capillary beds, leading to glomerulonephritis, polyarthritis, uveitis, and vasculitis. Secondary immune-mediated diseases are more commonly seen in FIV- than in FeLV-infected cats. When comparing plasma electrophoretograms, FeLV-infected cats do not show hypergammaglobulinemia and hyperproteinemia significantly more often than non-infected cats, whereas in FIV-infection, hypergammaglobulinemia and hyperproteinemia occur significantly more commonly [4,157].

Nevertheless, immune-mediated diseases have been described in FeLV-infected cats as well. While humoral immunity to specific stimulation decreases during the course of FeLV infection, nonspecific increases of IgG and IgM have been noted. The loss of T-cell activity in combination with the formation of antigen antibody complexes promotes immune dysregulation [158]. Immune-mediated diseases described in FeLV-infected cats include IMHA [159], glomerulonephritis [160], uveitis with immune complex deposition in iris and ciliary body [161], as well as polyarthritis [58]. Chronic progressive polyarthritis can be triggered by FeLV; in about 20% of cats with polyarthritis, FeLV seems to be an associated agent [58]. Measurement of FeLV antigen has shown that cats with glomerulonephritis have more circulating viral proteins than do other FeLV-infected cats. Antigens that can lead to antigen antibody complex formation include not only whole virus particles, but also free gp70, p27, or p15E proteins [162,163].

Immune-mediated diseases observed in FIV-infected cats are caused by an excessive immune response leading to hypergammaglobulinemia [4,104,164]. Hypergammaglobulinemia reflects polyclonal B-cell stimulation and is a direct consequence of FIV infection, because experimentally FIV-infected specific pathogen-free (SPF) healthy cats also develop hypergammaglobulinemia [164]. Increased IgG as well as circulating immune complexes have been detected in FIV-infected cats [165].

### 3.6. Stomatitis

Chronic ulcero-proliferative gingivostomatitis is very common in retrovirus-infected cats, especially in those with FIV infection. In cats naturally infected with FIV, it is the most common syndrome (affecting up to 50%). It characteristically originates in the fauces and spreads rostrally, especially along the maxillary teeth. Histologically, the mucosa is invaded by plasma cells and lymphocytes, accompanied by variable degrees of neutrophilic and eosinophilic inflammation. Lesions are often painful, and tooth loss is common. Severe stomatitis can lead to anorexia and emaciation. The cause of this syndrome is unclear, but the histologic findings suggest an immune response to chronic antigenic stimulation or immune dysregulation. Circulating lymphocytes of cats with stomatitis have increased expression of inflammatory cytokines [103], further implicating immune activation in the pathogenesis of this condition. This type of stomatitis is not always correlated with FeLV or FIV infection [166], and is usually not seen in SPF cats experimentally infected with FeLV or FIV, suggesting that exposure to other infectious agents also plays a role [167]. Concurrent feline calicivirus (FCV) infection is often identified in the oral cavity of these cats, and experimental and naturally occurring co-infection of FIV and FCV infection results in more severe disease [168,169].

## 4. Conclusions

FeLV can cause severe clinical syndromes, and progressive FeLV infection is associated with a decrease in life expectancy. Still, many owners still elect to provide therapy for their FeLV-infected cats, and with proper treatment, FeLV-infected cats, especially in indoor-only households, may live for many years with good quality of life. Diseases secondary to immunosuppression account for a large portion of the syndromes seen in FeLV-infected cats, and it is important to realize that many of these secondary diseases are treatable. In most naturally infected cats, FIV does not cause a severe clinical syndrome. Most clinical signs in FIV-infected cats reflect secondary diseases, such as infections and neoplasia, to which FIV-infected cats are more susceptible. With proper care, FIV-infected cats can live many years and, in fact, commonly die at an old age from causes unrelated to their FIV infection. While long-term studies describing clinical outcomes of naturally occurring FeLV and FIV infection are lacking, modalities for treatment of secondary infections or other co-incident diseases are available, and by treating these symptomatically, the life expectancy and quality of life of FeLV- and FIV-infected animals can be significantly improved.

**Table 2:** Comparison of clinical signs and their main pathomechanism in feline leukemia virus-(FeLV-) infected and feline immunodeficiency virus-(FIV-) infected cats

Clinical syndrome	FeLV	FIV
**Tumors**	62-times as likely as in non-infected cats, direct role of FeLV, mainly T-cell lymphoma	5-times as likely as in non-infected cats, indirect role of FIV, mainly B-cell lymphoma
**Bone marrow suppression**	common, anemia, thrombocytopenia, neutropenia, pancytopenia, primary infection of bone marrow precursor cells and stroma cells	rare, mainly neutropenia, soluble factors inhibiting bone marrow function
**Neurologic disorders**	rare, direct influence of the virus, lymphoma and neurotoxic effects (of FeLV envelope glycoprotein)	rare, direct influence of the virus (specific FIV strains), impairment of astrocyte function
**Immunodeficiency**	common, several mechanisms, e.g., replication of virus in all bone marrow cells (including neutrophils), changes in cytokine pattern	common, several mechanisms, e.g., decrease in CD4^+^ cells, changes in cytokine pattern
**Immune-mediated diseases**	rare, e.g., immune-mediated hemolytic anemia	sometimes, hyperglobulinemia common with immune complex deposition leading to e.g., glomerulonephritis and uveitis
**Stomatitis**	common, multi-factorial disease	very common, multi-factorial disease
